# An effectiveness-implementation hybrid type 1 trial assessing the impact of group versus individual antenatal care on maternal and infant outcomes in Malawi

**DOI:** 10.1186/s12889-020-8276-x

**Published:** 2020-02-10

**Authors:** Ellen Chirwa, Esnath Kapito, Diana L. Jere, Ursula Kafulafula, Elizabeth Chodzaza, Genesis Chorwe-Sungani, Ashley Gresh, Li Liu, Elizabeth T. Abrams, Carrie S. Klima, Linda L. McCreary, Kathleen F. Norr, Crystal L. Patil

**Affiliations:** 1 University of Malawi, Kamuzu College of Nursing, PO Box 415, Blantyre, Malawi; 2 Johns Hopkins University, School of Nursing, 525 North Wolfe Street, Baltimore, MD 21205 USA; 3 University of Illinois at Chicago, School of Public Health, 1603 W. Taylor Street (M/C 932), Chicago, IL 60612 USA; 4 University of Illinois at Chicago, College of Nursing, 845 S. Damen Avenue (M/C 806), Chicago, IL 60612 USA

**Keywords:** Antenatal care, Group healthcare, Maternal and newborn health, Preterm birth, Implementation, Fidelity, CFIR, Sub-Saharan Africa

## Abstract

**Background:**

Sub-Saharan Africa has the world’s highest rates of maternal and perinatal mortality and accounts for two-thirds of new HIV infections and 25% of preterm births. Antenatal care, as the entry point into the health system for many women, offers an opportunity to provide life-saving monitoring, health promotion, and health system linkages. Change is urgently needed, because potential benefits of antenatal care are not realized when pregnant women experience long wait times and short visits with inconsistent provisioning of essential services and minimal health promotion, especially for HIV prevention. This study answers WHO’s call for the rigorous study of group antenatal care as a transformative model that provides a positive pregnancy experience and improves outcomes.

**Methods:**

Using a hybrid type 1 effectiveness-implementation design, we test the effectiveness of group antenatal care by comparing it to individual care across 6 clinics in Blantyre District, Malawi. Our first aim is to evaluate the effectiveness of group antenatal care through 6 months postpartum. We hypothesize that women in group care and their infants will have less morbidity and mortality and more positive HIV prevention outcomes. We will test hypotheses using multi-level hierarchical models using data from repeated surveys (four time points) and health records. Guided by the consolidated framework for implementation research, our second aim is to identify contextual factors related to clinic-level degree of implementation success. Analyses use within and across-case matrices.

**Discussion:**

This high-impact study addresses three global health priorities, including maternal and infant mortality, HIV prevention, and improved quality of antenatal care. Results will provide rigorous evidence documenting the effectiveness and scalability of group antenatal care. If results are negative, governments will avoid spending on less effective care. If our study shows positive health impacts in Malawi, the results will provide strong evidence and valuable lessons learned for widespread scale-up in other low-resource settings. Positive maternal, neonatal, and HIV-related outcomes will save lives, impact the quality of antenatal care, and influence health policy as governments make decisions about whether to adopt this innovative healthcare model.

**Trial registration:**

ClinicalTrials.gov registration number NCT03673709. Registered on September 17, 2018.

## Background

Sub-Saharan Africa has the world’s highest maternal mortality ratio and a large proportion of people living with HIV [1]. Obstetric hemorrhage and hypertensive disorders, aggravated by HIV, account for many maternal deaths [2, 3]. There have been substantial declines in under-five mortality, but neonatal mortality remains a public health challenge. Preterm birth is the leading cause of neonatal death [4, 5]. Further, surviving premature infants suffer due to higher risk for later mortality and morbidity, neurodevelopmental impairment, developmental delays, and stunting [4–6].

Antenatal care provides an opportunity for life-saving monitoring, health promotion, and health system linkages through early detection and timely intervention [7–9]. In sub-Saharan Africa, however, provider shortages, resource stockouts, and disrespectful care translate into long wait times and short antenatal care visits at which there is inconsistent provisioning of essential services and health promotion [10–12]. Providers’ failure to deliver essential services along with low attendance after the intake visit combine to reduce opportunities for early detection and timely intervention [13, 14]. For example, pre-eclampsia, a leading cause of maternal and perinatal death, can be addressed with reliable detection through blood pressure monitoring and early recognition of dangers signs, but less than half of women have their blood pressure measured at each antenatal care visit [15, 16]. Health promotion is minimal, especially for HIV prevention, so exposure to HIV from untested partners is still a problem. Gaps persist in the continuum of HIV care for HIV-infected women and their infants during and after pregnancy [13, 14, 17]. When women can, they choose health facilities they perceive as having better quality of care because their healthcare experiences drive their care-seeking behavior; greater satisfaction is associated with attending more antenatal care visits [18–23]. Change is urgently needed to optimize the impact of antenatal care in sub-Saharan Africa.

To address antenatal care quality gaps, WHO recently revised their recommendations and doubled the number of contacts from 4 to 8 and to emphasize a positive pregnancy experience [22, 24]. How will low-resource health systems simultaneously double the number of visits and improve quality? A major paradigm shift in the current individual antenatal care model is needed. A promising model WHO identified as needing further exploration in the context of rigorous research is group antenatal care [24]. In group antenatal care, 8–12 women with similar gestational ages attend all of their visits together and see the same midwife over the course of the pregnancy.

CenteringPregnancy© is the only group antenatal care model with a large body of rigorous evidence supporting its effectiveness and the feasibility of bringing it to scale [25–27]. A two hour CenteringPregnancy group antenatal care visit includes self and midwife health assessments in a group space, interactive learning, and community building. Women measure their own blood pressure and weight, briefly consult the midwife in a corner of the room, and then meet for 80–90 min of interactive health promotion discussion enlivened by activities, games, and role-plays. Women form relationships with providers and one another as they collaboratively generate strategies to improve health across the pregnancy and into the early postpartum [25].

US-based randomized trials and a matched cohort study implemented with high fidelity to the model showed significant declines in prematurity rates and improved attendance, satisfaction, breastfeeding practices, safer sex behaviors, and uptake of family planning [26–30]. In one randomized control trial (RCT), higher levels of women’s engagement in interactive discussions related to a greater reduction of risk for prematurity [31]. When HIV and STI prevention was integrated into CP (termed CP+), women had increased condom use and fewer repeat pregnancies [28]. This body of research shows the power that the CenteringPregnancy model has to improve antenatal care. Group antenatal care fundamentally alters service delivery, allows for longer and woman-centered care, and has the potential to meet demands for higher quality care.

To expand the benefits of group antenatal care to sub-Saharan Africa, we adapted the only evidence-based group antenatal care model, CenteringPregnancy, for use in Malawi and Tanzania [32–36]. To ensure that the model was adapted with fidelity to CenteringPregnancy’s core components and associated practices and would be replicable, we consulted with the model’s developer, Sharon Rising. We then conducted a 2-arm randomized pilot in which pregnant women (*n* = 218) were randomly assigned to individual (usual) focused antenatal care or group antenatal care (intervention). Significantly more women in group than individual antenatal care completed ≥4 visits (94% vs 58%) and attended a postnatal visit (75% vs 50%). Rates of partner HIV testing were higher for those in group antenatal care (51% vs. 27%). Other positive outcomes included higher satisfaction, more HIV-related knowledge, and less mental distress. The showed that that group antenatal care can be offered with fidelity and that individual randomization was feasible and acceptable, supporting the need for a rigorous effectiveness trial [33].

In addition to our work, research conducted in Ghana [37] and Iran [38] were cited by the WHO to support group antenatal care’s potential for impact [24]. However, the WHO was cautious, because premature adoption of a new model can have serious negative consequences, as exemplified by the reversal of the 4-visit model [39]. A growing body of literature shows that group antenatal care is feasible and acceptable in many low and middle-income countries across the globe [40–50]. Additional work is underway in Mali, China, Ethiopia, Surinam, and elsewhere [51]. Most of these studies do not yet have results, but 3 studies in Africa have noted positive outcomes [43, 52, 53]. A large cluster-randomized trial in Nigeria and Kenya found greater antenatal care attendance in both countries and more birth planning for a facility-based birth in Nigeria [52, 53]. Two smaller studies in Nigeria with weaker designs reported greater attendance, greater knowledge of danger signs, and more use of health facilities for delivery [54, 55]. Importantly, these initiatives do not report sufficient detail about if and in what ways their models deviate from the evidence-based CenteringPregnancy model (i.e., fidelity). It is not clear if the evidence-base practices associated with the positive outcomes of CenteringPregnancy were retained in these adaptations [25]. Additionally, many studies lack adequate power to test for effects on birth outcomes (e.g., prematurity and/or low birthweight). One large cluster randomized trial currently underway in Rwanda is adequately powered to examine preterm birth, but the model deviates on three key practices of the CenteringPregnancy model (e.g., lack of continuity of co-facilitators and group members (women can drop into other groups) and group size can be larger than 12) [42, 43]. Our study, taking place in Malawi, fills both the power and fidelity gaps while implementing group antenatal care in the context of a midwife shortage and high rates of prematurity and HIV infection [13, 56, 57].

## Methods

### Study aims

#### Aim 1 (effectiveness)

Using a randomized controlled trial (RCT) with individual randomization, we evaluate the effectiveness of group antenatal care through 6 months postpartum. We hypothesize that compared to individual care, women in group care and their infants will have less morbidity and mortality and more positive HIV prevention outcomes, including:

**H1.** Fewer preterm births (*primary outcome*), stillbirths, low birthweight infants and neonatal and maternal mortality.

**H2.** Higher rates of HIV testing for partners (*primary outcome*) and women at first visit and in late pregnancy if HIV negative, and more HIV prevention knowledge and behaviors.

**H3.** More optimal secondary outcomes: healthcare utilization, satisfaction with care, pregnancy-related knowledge, healthy behaviors, mental distress, anemia, hypertension, postpartum bleeding (> 500 ml), exclusive breastfeeding, uptake of family planning and early repeat pregnancy.

**H4.** *Exploratory:* For the subset of ~ 130 HIV-infected women: received antiretroviral therapy (ART) from intake through 6 months postpartum and infant HIV status known.

#### Aim 2 (implementation)

We identify clinic-level degree of implementation success and contextual factors associated with success for each clinic and across clinics.

### Design

This study uses a hybrid type 1 design [58], to evaluate effectiveness and document implementation processes at six clinics in Blantyre District, Malawi.

### Study setting

The Malawi Ministry of Health is preparing to adapt and rollout the WHO model nationwide [24]. Although this 8-visit model is not yet the national standard of care, the Ministry of Health felt that to be equitable, our RCT should offer eight antenatal care contacts to women in both group and individual study conditions. To enhance generalizability, we selected rural, peri-urban, and urban clinics that maximized variability in client volume (the key factor affecting implementation in our pilot) and staffing in Blantyre District, Malawi (Table 1). The variability in these clinics will allow us to assess if government-run clinics can implement group antenatal care across the spectrum of everyday clinic conditions. We also ensure that each clinic has the same equipment available to both the control and intervention study conditions.

**Table 1:** Clinic staffing and volume

Clinic	Number of Midwives	New clients per month
1	3	40–50
2	7	80–90
3	15	50–60
4	11	80–90
5	12	85–90
6	16	200–250

### Aim 1 Study population (effectiveness)

The study population is comprised of pregnant women over age 14 and less than 24 weeks pregnant. Those under age 15, more than 24 weeks pregnant, or unable to make an informed choice (e.g. unable to converse about the study) are ineligible by design.

However, during the ethical approval process, our design had to be modified because of the recent change to the law forbidding marriage for women under the age of 18. At present, enrollment of minors raises the issue of needing to report the marriage as criminal. The US practice of issuing a certificate of confidentiality to avoid exposing research participants to legal jeopardy is not possible in Malawi. Until this issue is resolved the University of Malawi College of Medicine Research and Ethics Committee is not approving research with pregnant adolescents. We are actively seeking a strategy to allow inclusion of adolescents under age 18, but we do not know how long this limitation will continue.

#### Recruitment, consent, and baseline data collection

Regardless of study condition, each woman is assessed for eligibility after her first individual intake visit at which her health and gestational age are assessed, and laboratory and HIV tests are completed. Some of these data are used to determine eligibility (e.g., age, gestational age). Women then go through the informed consent process, sign the consent form, and then the baseline self-report survey. Refusals and reasons given by eligible women will be documented as indicators of self-selection bias.

#### Randomization

Using a computer program, our statistician generated randomization card sets. Each set contains randomized assignment for a cluster of 16 (8 to each study condition) or 24 women (12 to each study condition). A new packet is used with each new cluster. After completion of the baseline survey, the woman takes the next card and, until it is revealed, neither the woman nor researcher is aware of her assignment.

#### Retention

To maximize retention in the effectiveness evaluation across the 4 data collection time points, we use the same strategies that were successful in our pilot. When possible, we obtain cell phone numbers at which the woman can be reached as well as the location of her home. If a woman misses a data collection appointment and cannot be reached, a health surveillance assistant will go into the community and attempt to locate the woman.

#### Sample size and power

Sample size determination was based on power analyses for the two primary outcomes: rates of preterm birth and rates of HIV testing for women and partners (H1 & H2). At the writing of the proposal, the preterm birth rate for Malawi was estimated to be 18% [57]. We expect that the preterm birth rate for women in the individual antenatal care will mirror the national rate. Because a US-based RCT found a 30% reduction in the preterm birth rate for women in group antenatal care [26], we expect the preterm birth rate for women in group antenatal care in Malawi to reflect a 30% reduction. Our pilot data showed an attrition rate of 19% through 6 weeks postpartum; therefore, we assume a 30% attrition rate for a longer duration of 6 months follow-up. Recruitment and randomization are done in clusters and we assume a within-cluster correlation of 0.1; therefore, a sample size of 1776 will ensure 80% statistical power (two-sided alpha = 0.05) for detecting group preterm birth rate difference after attrition (final N at 6 months = 1244). For H2, our pilot results from Malawi showed that 51% of group antenatal care partners were tested during pregnancy versus 27% for usual care. The proposed sample size of 1776 will ensure a statistical power of > 99% for detecting similar difference. Power analyses for Aim 1 were performed via simulations in SAS for dichotomous outcomes in a multi-level design. The proposed sample of 1776 women at baseline (1244 after attrition) ensures > 80% statistical power for testing our secondary outcomes.

For our exploratory hypothesis (H4), participating clinic data for the first 6 months of 2017 showed an average HIV infection rate of 10.7%. With a sample of 1776 (1244 after attrition), we estimate there will be a subsample of 133 HIV-infected women at 6 months postpartum. Only 78.8% of women diagnosed at their first antenatal care visit are retained in care 12 months later; 60% of those who get their infant tested do not return for the infant’s results [59]. Our sample will not provide adequate power to determine differences in retention by model of care; however, clinically important information about group antenatal care’s effect on continuation of care for HIV-infected women and infant follow-up through 6 months postpartum will be beneficial.

### Aim 1 Effectiveness study conditions

The care for women in the RCT differs from national antenatal care because usual antenatal care (control condition) consists of 8 antenatal care contacts aligning with the 2016 WHO recommendations [24]. The postnatal care schedule is unchanged with the expectation of visits with 24 h, 1 week, and 6 weeks postpartum. Women, regardless of study condition, are offered the same intake visit and 8-visit antenatal care schedule. The two study conditions are described below:

- ***Control condition, Individual care***: Women listen to a health lecture and are provided antenatal and postnatal care services on a first come, first served basis. They meet individually with a midwife for a physical assessment. Women complete laboratory tests (including HIV testing) at their intake (first) visit.
- ***Intervention condition, Group care***: Women have the same number of visits as those in usual care at the same time points in their pregnancy and after delivery. The intake visit ends with assignment to group care and each woman is given her group appointment schedule. All subsequent antenatal care visits occur as a 2-h group visit with the same women and co-facilitators, one midwife and one community volunteer. The 1-week postpartum visit is an individual one because of the short interval between delivery and the recommended visit. Women in the group will deliver over the course of about 4 weeks, so it is not feasible to have a group visit at the 1-week time point. The second postnatal visit at 4–8 weeks after delivery is a 2-h group visit.

### Intervention: 3-step sequence for implementation

**Fig. 1:**
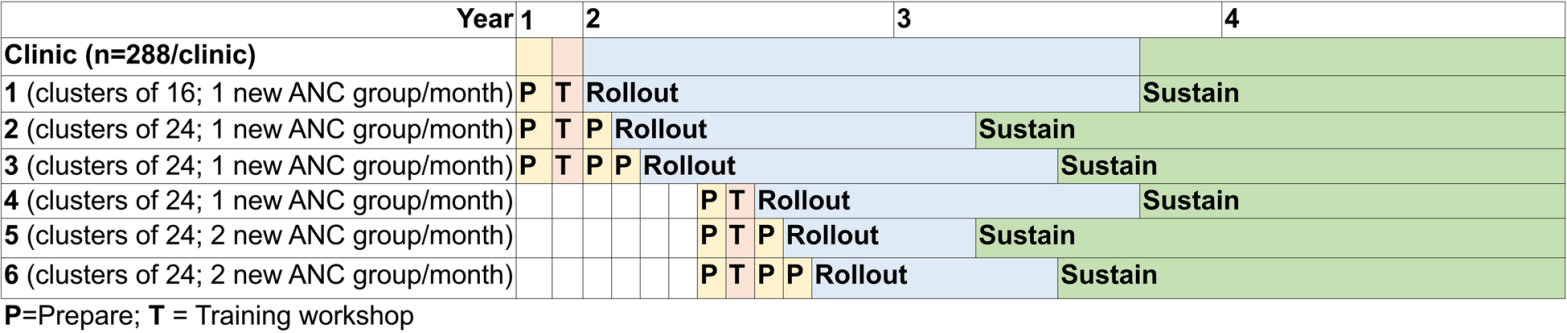
Timeline showing the 3-Step Implementation Model used by each clinic

Following a model that was used to successfully scale-up Kangaroo Mother Care in South Africa, each clinic uses a 3-step sequence for implementation: prepare, rollout, and sustain (6 months with support and 6 months independently). We purposely staggered rollout to ensure that our implementation team is able to provide intensive support and interactive assistance as each clinic begins to offer group antenatal care. The three steps are described in more detail in the Procedures section and summarized in Fig. 1.

#### Prepare (2 months)

Together with our implementation team, each clinic develops and presents a plan to fit their clinic’s context and sends future group care facilitators (providers and community volunteers) to a training workshop.

#### Rollout

Each clinic enrolls equal numbers of group and individual antenatal care participants in clusters for the effectiveness study (Aim 1).

#### Sustain (12 months)

With support and assistance from the implantation team, the clinic will review their experiences and the available evidence to decide whether to continue offering group antenatal care at their clinic.

### Intervention: group antenatal care implementation toolkit

Our evidence-based group antenatal care Toolkit provides clinics with all of the information and materials needed to implement group antenatal care [60–62]. The Toolkit includes a clinic implementation guide, group antenatal care training and facilitation guide, benchmarks, a training video, and interactive learning materials. Although the Toolkit can be used alone, research shows that implementation proceeds more quickly and with greater fidelity when supportive consultation and interactive assistance is provided [62–66]. To help with planning and resolving emerging challenges, our implementation team provides intensive and supportive consultation and interactive assistance each week as each clinic prepares and during the first 2 months of rollout of the group antenatal care model. We work with clinics as they adapt group antenatal care to their context (e.g. considerations may include staffing levels, busiest days, client preferences). We ensure that adaptions will not threaten fidelity. Thereafter, we meet monthly and then during *sustain*, quarterly. Clinics are welcome to call between meetings to discuss emergent problems. Experiences, challenges, and solutions will later be compiled and shared in a “Lessons Learned” chapter that will be added to the implementation guide.

Given there were no experienced group antenatal care trainers in Malawi, two experienced CenteringPregnancy trainers from the US led the 4-day training workshop. The workshops are appropriate for both midwives and the community volunteers with lower literacy and designed to provide opportunities for experiential learning and skills-building. After co-facilitators at clinics 1–3 have gained substantial experience, we will identify those who would like to become group antenatal care master trainers. The US trainers will then offer an Advanced Training Workshop in Year 3 to develop these skills. Master trainers will then gain experience because they will lead the Year 3 training workshop for clinics 3–6. This builds capacity since a local cadre of group antenatal care master trainers will be available to provide future trainings and implementation support.

### Measures

Aim 1 outcomes evaluating the impact of group antenatal care are measured at four time points (T1: baseline, T2: late pregnancy, and T3 and T4, 2- and 6-months postpartum). Data are produced from self-report surveys, health records extractions, and two biomarkers assessing anemia (T1-T4) and pregnancy status (T4). Survey measures were selected from those used in our pilot and most have been used with similar clinical populations and showed high reliability (Table 2; Additional File 1). Pregnancy knowledge and behaviors indices have been modified to reflect expansion from 4 to 8 visits and behavioral recommendations. These items were reviewed by a panel of expert midwives for content validity. Variables examined at 6 months after delivery are assessed using standard measures.

**Table 2:** Group antenatal care effectiveness outcomes (Aim 1)

Construct	Operational Measure	Time				Source^a^
		1	2	3	4	
H1. Less prematurity and mortality						
Preterm Birth (Prim)	< 37 weeks gestational age			•		H, S
Spontaneous abortion	Pregnancy loss < 20 weeks			•		H, S
Stillbirth	Baby born with no signs of life ≤28 weeks gestation [67]			•		H, S
Low birthweight	< 2500 g, measured within 24 h of birth			•		H, S
Neonatal death	Newborn dies with 28 days after birth			•	•	H, S
Maternal mortality	Death in pregnancy or ≤ 42 days of end of pregnancy		•	•		H, S
H2. More positive HIV prevention outcomes						
Partner HIV Test (Prim)	Proportion of partners tested during pregnancy		•			S
Women, HIV Test	Initial HIV test; If seronegative, repeated in 3rd trimester	•	•			
HIV Knowledge	General knowledge (5 items)	•	•			
PMTCT Knowledge	Mother-to-child transmission (4 items)	•	•			
Sexual Health Behaviors	Condom use; Partner communication (5 yes/no items)	•	•	•	•	S
H3. Optimal secondary outcomes						
Antenatal & postnatal care	Attendance; birth in a health facility (yes/no)			•		H, S
	21 services received; 18 educational topics *(modified)*		•			
Satisfaction with Care	10-items; 5-point Likert scale; Range 10–50, α =0.980		•			S
Pregnancy Knowledge	25 items based on content (*modified for this study)*	•	•			S
Healthy Behaviors	14 recommended behaviors *(modified for this study)*	•	•			S
Mental Distress	Self Reporting Questionnaire [68–71]; 20 items; α = 0.789	•	•	•	•	S
Anemia	Hemoglobin (Hb), HemoCue® (Hb < 11//dL) [72]	•	•	•	•	T
Hypertension	Blood pressure changes and symptoms	•	•	•		H, S
Postpartum bleeding	Bleeding (> 500 ml); hemorrhage (> 1000 ml)			•		H, S
Exclusive Breastfeeding	Exclusive breastfeeding duration, # days				•	S
Family Planning	Using a family planning method				•	S
Early repeat pregnancy	Negative test and no reported pregnancy loss				•	S, T
H4. Exploratory: Successful transition in continuum of HIV care (subset of HIV-infected women)						
Women	Received ART medication	•	•	•	•	S
Infant	Infant HIV test, status known			•	•	H, S

^a^Source: *H* Health record, *S* Self-report survey, *T* Medical test

### Aim 2 Study population (implementation)

Data for the implementation portion of the study are drawn from key stakeholders at the Ministry of Health, District Health Office, clinic administration, and co-facilitators (antenatal care midwives (and master trainers) and community volunteers). Eligibility criteria are based on the person’s position at the time of data collection (e.g., Director of the Reproductive Health Unit, midwife at participating clinic). Consent is obtained before data are collected for the first time. Persons no longer in the designated position are dropped, and persons coming into these positions are consented to participate at their first data collection.

Our Aim 2 degree of implementation success measures are in Table 3. Three indicators (continuation, reach and fidelity) are assessed after 12 months of sustaining. For clinics that continue to offer group care, degree of success is examined using a combination of reach and fidelity indicators and the relationship between reach and fidelity will be explored in our analysis. We use the same fidelity observation procedure and data collection instrument used in our pilot (Additional File 2). Guided by the 5 domains of the consolidated framework for implementation research (CFIR), contextual factors are events, situations, and clinic contextual factors that are abstracted from study notes, interview data, and staff surveys (Additional file 3) [73, 79, 80].

**Table 3:** Measures and data sources for degree and variation of implementation success (Aim 2)

Indicators based on data from the last quarter of sustain independently		Source
Continuation	Yes/No indicating ceased or continued to offer group antenatal care	Benchmarks
Reach	% in group care based on the total number of antenatal care clients	Benchmarks
Fidelity	Quarterly clinic mean scores; High fidelity indicated by high group engagement, high session management and interpersonal facilitation skills (e.g., controlling negativity, drawing out participants), and overall rating as more like discussion than classroom.	Observations
Contextual Factors by CFIR Domain [73] (ongoing)		
Intervention	• Time: Group vs. usual time required to deliver antenatal care	Interviews Study Notes
	• Additional group care expenses: training, equipment and supplies	
	• Other: factors affecting implementation (space, scheduling)	
Outer Setting	• Ministry of Health (annually); District Health Office (annually); Safe Motherhood Task Force (Quarterly); Other stakeholders, as needed	Meetings Interviews
Inner Setting	• Clinic characteristics and events reported (staff transfers, leadership, interactions) • Successes, challenges, solutions, decisions, interpersonal interactions, and evaluation of evidence; coded as positive, negative or mixed	Benchmarks Interviews Observation
Individual (Staff)	• Basic demographics, including education level and experience	Survey
	• Maslach Burnout Inventory (9 items; α > .79) [74–76];	
	• Health Worker Motivation (10-item; range 10–50) [77, 78];	
	• Perspectives on group care (5 items) at baseline and beginning and end of *sustain*	
Process	• Quarterly Benchmark Scores: 25 yes/no items (1 point for each yes). Integrates co-facilitators’ self-evaluation of fidelity and time records. Patterns over time: Fail; Interrupted; Sporadic; Continuous	Benchmarks Self-evaluations
	• Fidelity: Quarterly ratings	Observations

### Procedures

As described above, each clinic follows the same sequence of steps to Prepare, Rollout and Sustain (6 months with support, 6 months independently) group antenatal care. We staggered the initiation of group antenatal care to allow the implementation team (not blinded to study condition) provide intensive Interactive Assistance. During rollout, the effectiveness team (blinded to study condition) leads the collection of effectiveness data at the four time points during pregnancy (T1 and T2) and postpartum (T3 and T4).

### Aim 1 Effectiveness procedures

Clusters of 24 women are currently being enrolled with 12 randomly assigned to each study condition (Fig. 1). The clinic with the lowest patient volume enrolls cohorts of 16 women with 8 assigned to each study condition which extends their time to complete rollout. Before randomization and to promote privacy and better reporting of sensitive information each woman completes a baseline self-report survey administered off-line using ACASI [81].

To manage timely data collection, a spreadsheet lists all participants as they enroll, along with ID numbers, clinic, contact information, enrollment date, and weeks gestation at entry. After each day’s data collection, the team enters the date information was obtained or, if planned data collection was not completed, the reason (e.g., no show, in-hospital etc.) and planned follow-up action and date. Each week the list of women needing follow-up, type of follow-up and their contact information is generated to guide the data collection schedule. At weekly effectiveness team meetings, recruitment plans and the spreadsheet identifying follow-up data collection needs is presented. The team reviews this and makes assignments. The team discusses challenges; ideas for reaching women lost to follow-up are discussed; and plans for the next week are made.

A team member calls each woman to schedule the data collection appointment and offer reminders. If a woman does not have a cell phone, health surveillance assistants from the clinic will go to the woman’s home to make the appointment. Women’s hemoglobin (Hb) levels are tested at all 4 time points using a simple point-of-care blood droplet test, following standard procedures provided by the manufacturer to ensure validity [72]. At the 4th survey, each woman is asked to take the pregnancy urine test kit in a clinic restroom. A team member will review the results with her and record the result. We discard all biomarkers materials in our trash container, which is removed at the end of the day and disposed of in the clinic incinerator. For women who were identified as HIV+ at intake or during pregnancy, maternal and infant HIV clinic attendance and infant HIV status at 6 months will be verified from medical records. Shortly after the expected delivery date or early loss, our data clerks will work with clinic staff to search delivery records to verify fetal loss, stillbirth, or live birth plus prematurity and birthweight. Most women deliver at the clinic where they obtain care, but if a woman has complications in pregnancy or delivery, she is sent to a referral hospital. After a woman gives birth, a research team member will obtain these data to capture the birth outcomes from records. Hb values, pregnancy test results, and health records will be recorded by the research team in the participant’s Health Passport (as appropriate) as well as on data collection forms developed for the research project, using the participant’s project ID code.

### Aim 2 Implementation procedures

As described above, over the course of 3 years, each of the six clinics will prepare, rollout, and sustain group care. Rollout is staggered to ensure that the implementation team can provide intensive support and interactive assistance. The procedures of the 3 steps of the implementation model are described in detail below.

#### Prepare (2 months)

Our implementation team sensitizes each clinic to group antenatal care. Each clinic conducts a group antenatal care walk-through, identifies who will coordinate group antenatal care, identifies community volunteers, adapts the plan to fit their clinic’s context, presents their plan at a clinic meeting, and sends providers and community volunteers who will be co-facilitators to a group antenatal care training workshop.

*Rollout* is the period in which the Aim 1 Effectiveness component is conducted. Each clinic enrolls equal numbers of group and individual antenatal care participants in clusters for the effectiveness study. The evaluation team completes consent, baseline data collection, and random assignment and then provides the clinic with the lists of women randomly assigned to group antenatal care. During rollout, providers can call our team for advice as needed to address any unforeseen barriers/issues.

*Sustain* is a 12-month process that begins with a clinic decision to continue offering group antenatal care during the month that the last cluster of women is enrolled in the effectiveness study. The co-facilitators’ experience and feedback is presented to clinic administration and staff so that decisions can be made about whether to continue offering group antenatal care. If so, they also determine if they need to train additional facilitators and the number of groups they will enroll each month. To build capacity, we treat the first 6 months of *sustain* as transitional and continue to offer our support and assistance. The clinic continues to receive interactive assistance from the implementation team and completes benchmarks each quarter. If they decide not to enroll new groups, they complete care for women already enrolled and cease participation.

After another 6 months, the clinic will again review their experiences and the available evidence to decide whether to continue offering group antenatal care at their clinic. Clinics will continue to complete and share their benchmarks, and an implementation team member will make a brief quarterly check-in to pick up a copy of the benchmarks and consult. If training is needed, the clinic will contact a Malawian master trainer to arrange. Some clinics may elect to not continue offering group antenatal care and others may flounder and find themselves unable to continue. Thus, some clinics may not complete 12 months of sustaining. We will assess their ‘end point’ as when they no longer form groups.

### Data analysis

#### Data management

Project protocols promote proper and timely preparation of data for analysis and secure data storage. All data are identified by location, date and clinic. A unique individual code is assigned to individual-level data. Signed consent forms and a master list linking names and code numbers are securely stored in locked cabinets separately from data. Data are transferred to UIC using secure cloud storage and strict confidentiality guidelines for HIPAA compliance and data confidentiality guidelines for Malawi.

#### Periodic clinic reports

The 6 clinics roll out at different time points and require their own clinic data so they can assess the impact of group antenatal care for their own clients. Every 6 months, our team will generate a descriptive report summarizing outcomes for group and individual care by each clinic and totals for all clinics, using the cumulative data available. Reports will also be shared with the Ministry of Health’s Safe Motherhood Task Force.

#### Aim 1 Statistical analyses

The goal is to evaluate the overall effectiveness of group antenatal care compared to individual care. In preliminary analyses, we will complete a CONSORT diagram of recruitment, retention, and loss to follow-up from initial recruitment through final data collection. We will determine retention rates and examine attrition bias, e.g., significant demographic differences between those retained and those lost to follow-up using Cox proportional hazards regression for time-to-dropout. Randomization success will be evaluated by comparing group differences in baseline demographics and measures of interest such as HIV testing, pregnancy knowledge and behaviors, etc. Any significant differences in sites’ demographic characteristics at baseline and attrition biases will be controlled in later analyses. Bivariate analyses between the outcome variables and study group will first be conducted using t-tests (continuous variables) or Chi-squared tests. Potential clinical site differences will be identified using Analysis of Variance (ANOVA) models for continuous variables or Chi-squared tests for categorical variables.

All multivariate analyses will be conducted using multi-level hierarchical models, with clinics entered in models as both fixed effects (to adjust for mean site differences) and random effects (to account for within-site correlations). For outcomes that are measured only once, mixed-effects linear regression (continuous), Poisson regression (count data), or logistic regression (dichotomous or ordinal) models will employed. Intra-class (site) correlation coefficient will be estimated. Group assignment, sites, women’s demographics, and obstetric characteristics will be included as fixed effects. Potential effect modifiers will be identified (based on theoretical reasons) by testing the interaction between group and the potential modifier. For continuous outcomes that are measured more than once, such as mental distress or partner communication, mixed-effect regression models will be employed to examine group effect over time with both random site and individual (nested within site) effects. Generalized Estimating Equation models will be employed to analyze dichotomous repeated outcomes (such as consistent condom use in the last 2 months). In all repeated measures models, the interaction between group and time will be the parameter of interest, and time-point specific group difference will be estimated from the model. In all multivariate models, backward selection methods will be employed to select significant factors that are associated with the outcome. All statistical tests will be two-sided tests, controlling for probability of Type I error of 0.05.

#### Aim 2 Mixed-methods analyses

Addressing Aim 2 is an iterative process that begins early and continues throughout the study. We extract contextual factors (events or statements) from study notes, interviews, and observations. We focus on reported successes, challenges, solutions tried and whether successful, as well as champions and naysayers. We will then categorize events or statements according to CFIR domains and constructs.

Data will be coded separately by 2 coders who will consult with the team to review discrepancies, refine code definitions, and recode until intercoder reliability exceeds 85%. In addition to the CIFR, new themes may emerge; these will be developed into new codes and discussed in team meetings. Final codes will be compiled in the master codebook and applied to the coding of qualitative data [79], [82].

From the last quarter of *sustain* for each clinic, we will determine the degree of success using 3 indicators (Table 3). We will use data from the last quarter of *sustain*, because this time interval reflects each clinic’s performance when sustaining independently. The minimum indicator of implementation success will be whether a clinic continues to offer group antenatal care or not. For clinics that continue, we will base degree of success on a combination of reach and fidelity and compare clinics using analysis of variance. Together these indicators will allow us to categorize the 6 clinics according to their degree of success. We cannot know what this will look like beforehand, but we anticipate 2 or 3 major categories (e.g. successful or not, or high, medium, and low).

As each clinic completes the *sustain* step, we will begin to describe implementation processes and factors related to prepare, rollout and sustain and their associations with degree of implementation success. Using a team-based case study approach, we will rate each construct according to whether it facilitated, hindered, or did not affect implementation [79]. The end product will be a case summary for each clinic [83, 84]. Our quantitative measures (i.e., staff burnout and motivation and fidelity scores) will first be analyzed using standard statistical procedures. For mixed methods analyses, these quantitative measures will be transformed into interval or dichotomous categories.

Using well-established mixed methods analyses, [85, 86] we will then compare across cases to identify constructs most strongly related to degree of implementation success (variable-oriented approach) [84]. Our mixed method analyses to construct the final across case matrix will also be guided by the procedures used by Damschroder and Lowery to identify contextual factors associated with greater or less implementation success for an obesity program in the US [83]. At the conclusion of Aim 2 analyses, we will identify the contextual factors that consistently relate to degree of implementation success (or failure) across all 6 clinics.

### Study status

At the time this manuscript was submitted for publication, the study was underway. Three clinics have completed the prepare step. The first group antenatal care training workshop was successful; 38 midwives and 9 community volunteers were trained. A total of 17 clusters of women are enrolled from three clinics (*n* = 352).

## Discussion

This is the first individually randomized RCT of group antenatal care in a low-income African country that has high fidelity to CenteringPregnancy, the only evidence-based group antenatal care model, and incorporates the 2016 WHO recommendations for eight antenatal contacts with woman-centered care. At the conclusion of this five-year study, we will be able to assess whether an eight-contact model meets women’s needs and if group antenatal care is equal to or better than the usual individual care model.

In addition to meeting WHO’s call for the rigorous study of group antenatal care, our effectiveness-implementation hybrid type 1 design also allows us to make an important contribution to implementation science. We incorporate process evaluation within the randomized trial so that we can systematically identify factors that impede and facilitate successful implementation over time. Implementation of an evidence-based model with fidelity (i.e., adherence to and high-quality delivery of the core components) can be difficult in the best of circumstances. Midwives in Malawi and other poorly-resourced health systems with a shortage of providers may lack the capacity for evidence-based implementation [87]. We are facilitating replication through careful attention to fidelity, which is supported by the training workshop, an implementation toolkit, and technical assistance. We will document if implementation of the group antenatal care model at each clinic and over time is faithful to core components of the intervention. These data will be of interest to those implementing other evidence-based interventions in low-resource healthcare settings [88].

Although the well-established CIFR has been used once retrospectively in a low-resource setting, to the best of our knowledge, this is the first study to use CFIR prospectively to describe implementation factors in a low-resource country [73]. Findings from this evaluation will contribute to the growing body of evidence identifying key factors positively and negatively affecting implementation, with special relevance for low-resource settings. If effectiveness is demonstrated, the implementation evaluation will be critical to and facilitate national scale-up this paradigm-changing and transformative model that aims to improve antenatal care quality.

Group antenatal care has been shown to improve the quality of care and maternal and infant outcomes in the US. The implementation of group antenatal care in a context of high mortality and HIV prevalence is especially innovative because this is a comprehensive intervention that simultaneously addresses the multiple needs of women. In addition to saving lives by reducing rates of preterm birth and low birth weight, data show that investing in quality antenatal care provides a return on investment through health expenditure savings. In Malawi and many other lower income countries, nearly every woman will attend antenatal care several times over her life. Half of Malawian women have begun childbearing by age 19, and women have an average of 4.4 births [89]. If, in this context, group antenatal care can reduce risk for preterm birth, impact HIV prevention, including reducing new infections and prevention of mother-to-child transmission, improve infant feeding practices, and increase birth spacing, the population health impact will be enormous. If effective, it is also possible that higher quality of care may increase attendance and/or overload a clinic if the demand for services increases. Policy makers will have to consider the long-term population health cost savings that accrue from having healthier mothers, infant, and families and find ways to creatively support the education of more midwives to optimize antenatal care. This study will provide rigorous evidence documenting whether group antenatal care is effective and should be scaled up. If our study shows positive health impacts in Malawi, the results will provide strong evidence and valuable lessons learned for widespread scale-up other low-resource settings.

## Supplementary information

**Additional file 1:** Survey items and associated time points

**Additional file 2:** Fidelity and Self-Evaluation Forms

**Additional file 3:** Benchmark Scoring Sheet

## Data Availability

De-identified survey data will be made available by emailing a request to: cpatil@uic.edu. We will not provide full transcriptions of qualitative data as these may contain information that could compromise identity.

## Abbreviations

ACASI: Audio Computer-Assisted Self-Interview software
CFIR: Consolidated Framework for Implementation Research
RCT: Randomized Controlled Trial
WHO: World Health Organization
