# Supplementary material for: An effectiveness-implementation hybrid type 1 trial assessing the impact of group versus individual antenatal care on maternal and infant outcomes in Malawi
Source: BMC Public Health. 2020 Feb 10;20:205. doi: 10.1186/s12889-020-8276-x (PMC7008527; doi:10.1186/s12889-020-8276-x)
Supplement: Supplementary file 1 — Additional file 1: Survey items and associated time points [file 12889_2020_8276_MOESM1_ESM.pdf]

Survey data collection tool and time points

(T1 = baseline, T2 = late pregnancy, T3 = 2 months postpartum and T4 = 6 months postpartum)

Time Point				Section 1: Introduction
**T1**	**T2**	**T3**	**T4**	
1	1	1	1	Today's Date
1	1	1	1	Assistant's initials
1	1	1	1	Please enter the participant's ID code number
1	1	1	1	Please enter the participant's ID code number again
1	1	1	1	Survey time point
1	1	1	1	Are you continuing with an interview that was not completed before?
1	1	1	1	Clinic name
1	1	1	1	How old are you?
1	1	1	1	Village Name:
1	1	1	0	Describe an easy way to locate your home should a fieldworker from this study need to find you (street name, popular name of area, crossroads, etc.)
1	1	1	0	Do you have a cell phone?
1	1	1	0	Cell number 1
1	1	1	0	Cell number 2
1	1	1	0	Do you have access to the cell phone of a confidant, next of kin, or spouse?
1	1	0	0	Cell number 1
1	1	1	0	Cell number 2
1	1	1	1	Input Hb level
1	1	1	0	In total, how many times have you been to ANC clinic during this pregnancy?
**The following questions are about your health and your reproductive history.**				
1	0	0	0	In total, how many children have you given birth to?
1	0	0	0	Before this pregnancy, did you have any pregnancy losses, such as a miscarriage, abortion, or a stillbirth?
1	0	0	0	We are sorry to hear that you experienced loss. In total, how many miscarriages, abortions and/or stillbirths did you experience before this pregnancy?
1	0	0	0	Is this your first pregnancy?
1	0	0	0	Before this pregnancy, have you ever been pregnant with twins or triplets?
1	0	0	0	For any of your births, did you have a Cesarean section?
1	0	0	0	How many of your babies were born more than 3 weeks before your due date?
1	0	0	0	How many of your babies weighed less than 2.5kg at birth?
0	0	0	1	What is the result of the pregnancy test?
0	1	1	0	During this pregnancy, did you have pre-eclampsia?
0	0	1	0	During this pregnancy, did you have eclampsia
0	0	1	1	Since your new baby was born, have you had a checkup at clinic for yourself?
0	0	1	1	Since your new baby was born, how many postnatal checkups have you had?

Section 2: Health Records					
**T1**	**T2**	**T3**	**T4**	**Question**	**Response**
1	1	0	0	Passport	1. Yes<br>2. No
1	1	0	0	LMP	1. DD MM YY
1	1	0	0	EDD	1. DD MM YY
1	1	0	0	Deliveries	a. 1<br>b. 2<br>c. 3<br>d. 4<br>e. 0<br>f. 5+<br>g. Missing

1	1	0	0	Abortions	a. 0<br>b. 1<br>c. 2<br>d. 3+
1	1	0	0	Stillbirths	1. N<br>2. Y<br>3. Missing
1	1	0	0	C-sections	1. N<br>2. Y<br>3. Missing
1	1	0	0	Vacuum extractions	1. N<br>2. Y<br>3. Missing
1	1	0	0	Symphysiotomy	1. N<br>2. Y<br>3. Missing
1	1	0	0	Hemorrhage	1. N<br>2. APH<br>3. PPH<br>4. Missing
1	1	0	0	(Pre) eclampsia	1. N<br>2. Y<br>3. Missing
1	1	0	0	Height	1. 150+ cm<br>2. <150cm<br>3. Missing
1	1	0	0	Multiple pregnancy	1. N<br>2. Y<br>3 = Missing
1	1	0	0	Syphilis Date	DD MM YY
1	1	0	0	Syphilis Result	1. Positive<br>2. Negative<br>3. Missing
1	1	0	0	HIV Test Date	DD MM YY
1	1	0	0	HIV Result	1. Positive<br>2. Negative<br>3. Missing
1	1	0	0	Planned Delivery Place	1. N<br>2. Y<br>3. Missing
1	1	0	0	Plan for Transport	1. N<br>2. Y<br>3. Missing
1	1	0	0	How many visit dates?	__ __ (number)
1	1	0	0	How many Gest. Age?	__ __ (number)
1	1	0	0	How many Fundal height?	__ __ (number)
1	1	0	0	How many Position?	__ __ (number)
1	1	0	0	How many Fetal heart?	__ __ (number)
1	1	0	0	How many Weight (kg)?	__ __ (number)
1	1	0	0	How many BP?	__ __ (number)
1	1	0	0	How many Urine prot.?	__ __ (number)
1	1	0	0	How many times was she given SP?	__ __ (number)
1	1	0	0	How many times was she given FeFo?	__ __ (number)
1	1	0	0	Is she on CPT?	1. N<br>2. Y<br>3. Missing
1	1	0	0	Is she on ART?	1. N<br>2. Y<br>3. Missing

1	1	0	0	How many TTV vaccines did she get? See page 2 of the health passport.	__ __ (0-9)

Time Point				Section 3: Demographic and Socioeconomic Situation
**T1**	**T2**	**T3**	**T4**	**The next set of questions are about your household including your education and household situation**
1	0	0	0	What is your tribe?
1	0	0	0	What is your religion?
1	0	0	0	Have you completed primary school?
1	0	0	0	How many years of primary school did you complete?
1	0	0	0	Do you have an MSCE?
1	0	0	0	How many years of secondary school did you complete?
1	0	0	0	How many years of college or university did you complete?
1	0	0	0	Are you a subsistence farmer?
1	0	0	0	Are you regularly employed, meaning someone else pays you wages for full or part-time employment?
1	0	0	0	Do you do income-generating activities or piecework to earn your own money?
1	0	0	0	Over the last year, did your household ever run out of food or money to buy food?
1	0	0	0	Does your household have electricity or a generator?
1	0	0	0	Does your household own any farm animals or poultry?
1	0	0	0	Does any member of your household own agricultural land?
1	0	0	0	About how much land do members of your household own (specify number)<br>Mostly will know in acres
1	0	0	0	Think about the income generated by **you** and **all members** of your household for an average week. Approximately about how much money is made by all members of your household in an average week?

Section 4: Knowledge about Pregnancy (True/False)				
**Next, you will hear five different symptoms that pregnant women might experience. For each one, select "true" if you think this symptom is a danger sign and requires that a pregnant woman go to the health facility. Select "False" if you think the symptom is not a danger sign and does not require a woman to go to a health facility.**				
1	1	0	0	True or False? Ruptured membranes are a danger sign
1	1	0	0	Vaginal bleeding is a danger sign
1	1	0	0	Swelling of the feet is a danger sign
1	1	0	0	Swelling of hands and face is a danger sign
1	1	0	0	Severe headache is a danger sign
**The next set of questions will be about pregnancy and what you know about pregnancy.**				
1	1	0	0	True or False? Steady weight gain in pregnancy can help you have a healthy baby
1	1	0	0	Your blood pressure should increase a lot at the end of your pregnancy
1	1	0	0	Taking malaria pills once in pregnancy will prevent you from getting malaria in late pregnancy
1	1	0	0	It is normal to experience a sudden decrease in activity or movement by the fetus/baby
1	1	0	0	A spoonful of ndiwo (green leafy vegetables, beans, egg or other relish) contributes more to your health, such as your eyesight or resistance to disease, than a spoonful of nsima
1	1	0	0	Orange fanta is just as healthy for your health as a fresh orange
1	1	0	0	Papaya is healthier for you than biscuits
1	1	0	0	You need to eat the same amount of food as a woman who is not pregnant
1	1	0	0	A cup of milk provides more energy for work each day than a cup of water
1	1	0	0	You or your baby can be harmed if you take medications from the pharmacy or healer, without checking with your nurse/midwife
1	1	0	0	Being in the room while others are smoking cigarettes or marijuana is harmful for you and your baby
1	1	0	0	If you think your baby is not getting enough breastmilk, you should breastfeed more
1	1	0	0	All babies know how to breastfeed correctly, so you don't need to help the baby get latched onto the breast

1	1	0	0	On a hot day, you need to give your 2-month old baby water to drink
1	1	0	0	Your baby feels warm to the touch, but is not crying, so you do not need to take the baby to the clinic
1	1	0	0	Waiting 2 years before getting pregnant again is better for your health.
1	1	0	0	Using injections for birth control protects against both pregnancy and sexually transmitted diseases, including HIV.
1	1	0	0	If you are unable to find joy in any activity, you may be depressed.
1	1	0	0	You are depressed if you are feeling sad once in a while
1	1	0	0	How many months old should a baby be when they start drinking liquids or foods other than breastmilk, including water?
**The next questions are about STIs and HIV knowledge. Decide if the statement is true or false**				
1	1	0	0	True or False? Syphilis, gonorrhea, chlamydia, and trichomoniasis are sexually transmitted diseases that can be treated at hospital or clinic.
1	1	0	0	If your partner is diagnosed with a sexually transmitted disease, you need to get treatment at a clinic even if you do not have any symptoms.
1	1	0	0	It is possible to become infected with a sexually transmitted disease, including HIV, even if you only have sex with your partner
1	1	0	0	If you have a negative HIV test early in your pregnancy, you do not need to get another test during pregnancy
**Next, you will hear a series of questions about STIs and HIV, please answer yes or no.**				
1	1	0	0	**Yes or no?** Can the risk of HIV transmission be reduced by having sex with only one uninfected partner who has no other partners? Would you say yes or no?
1	1	0	0	Can a person reduce the risk of getting HIV by using a condom every time they have sex?
1	1	0	0	Can a healthy-looking person have HIV?
1	1	0	0	Can people get the AIDS virus from mosquito bites?
1	1	0	0	Can a person get the AIDS virus by sharing food with a person who has AIDS?
1	1	0	0	Is it possible to have a baby that is HIV negative even if both parents are HIV positive?
1	1	0	0	Can the virus that causes HIV/AIDS be transmitted from a mother to her baby during pregnancy and childbirth?
1	1	0	0	Can the virus that causes HIV/AIDS be transmitted from a mother to her baby by breastfeeding?
1	1	0	0	Are there any medications that a doctor or nurse can give a woman infected with the HIV/AIDS virus to reduce the risk of infecting the baby?

T1	T2	T3	T4	Section 5: Behaviors (Yes/No)
1	1	0	0	Did you sleep under a bed net last night?
1	1	0	0	Have you talked to your midwife about where you plan to deliver?
1	1	0	0	In preparation for birth, have you obtained the following supplies, including chitenge, razor, and pads, plastic sheet/mackintosh, basin etc.
1	1	0	0	In preparation for birth, have you decided how you will get to the health facility?
1	1	0	0	In preparation for birth, have you set aside money?
1	1	0	0	In preparation for birth, have you identified a blood donor?
1	1	0	0	Did you take your iron tablets yesterday?
1	0	0	0	Since you became pregnant, have you used any tobacco products (cigarettes, chewing tobacco, etc.)?
0	1	0	0	Since coming to ANC, have you used any tobacco products (cigarettes, chewing tobacco, etc.)?
1	0	0	0	Since you became pregnant, have you drunk alcohol or homebrew?
0	1	0	0	Since coming to ANC, have you drunk alcohol or homebrew?
1	0	0	0	Since you became pregnant, have you used marijuana or other drugs?
0	1	0	0	Since coming to ANC, have you used marijuana or other drugs?

1	1	0	0	This question is about moderate physical activity. By moderate physical activity we mean walking briskly, gardening or farming activities, and carrying heavy items such as water. In an average week, do you do moderate physical activity for a least a half an hour for at least 3 days a week OR a total of 1.5 hours or more doing moderate physical activity every week?
**Now you will hear 8 questions are about eating and the foods you may have eaten yesterday. Think about everything you ate yesterday, including snacks?**				
1	1	0	0	Yesterday, how many main meals did you eat?
1	1	0	0	Yesterday, about how many times did you snack or eat between main meals?
1	1	0	0	Yesterday, did you eat at least 3 fruits or vegetables?
1	1	0	0	Yesterday, did you eat any chicken, fish, eggs, insects or other meat?
1	1	0	0	Yesterday, did you eat groundnuts or groundnut powder, dried beans or peas, or soya pieces?
1	1	0	0	Yesterday, did you eat any green leafy vegetables, including wild/foraged leaves?
1	1	0	0	Yesterday, did you eat any yellow or orange fruits or vegetables, such as pumpkin, sweet potato, or ripe mango?
1	1	0	0	Yesterday, did you eat or drink milk (fresh or fermented) or yogurt?
**The following questions are about your relationships and your partner.**				
1	1	1	1	What is your marital status?
1	1	1	1	How long have you been in this relationship?
**The next 7 questions focus on communicating with your husband/partner**				
1	1	1	1	In the last two months, have you talked to your partner about eating a variety of foods and needing to eat more food during pregnancy and breastfeeding?
1	1	1	1	In the last two months, have you talked to your partner about exclusive breastfeeding?
1	1	1	1	In the last two months, have you talked to your partner about difficult subjects?
1	1	1	1	In the last two months, have you talked with your partner about family planning?
1	1	1	1	In the last two months have you talked to you partner about having an HIV test?
1	1	1	1	In the last two months have you talked with your partner about using a condom?
1	1	1	1	In the last two months, have you talked with your partner about being faithful to each other?
**Now you will hear a series of questions that focus on sexual health and prevention of HIV.**				
1	1	1	1	In the last two months, including your husband/partner you live with now, how many different people have you had sexual relations with, even if it was just one time?
1	1	1	1	In the last two months, when you have had sexual relations, how often did you use a condom?
1	1	1	1	Now think about the **most recent time you had sexual relations.** Did you use a condom the very last time you had sexual relations?
1	0	0	0	Have you had an HIV test in the past 12 months?
1	1	0	0	Did you get an HIV test during this pregnancy?
0	1	0	0	Did you have two HIV tests during this pregnancy (one at the beginning and one more recently?
0	0	0	1	Since your baby was born, have you had an HIV test?
1	1	0	0	In the last 12 months, did your husband/partner have an HIV test and shared the results with you?
1	1	0	0	During this pregnancy, did your husband/partner have an HIV test and share the results with you?
0	0	1	1	Since your baby was born, did your husband/partner have an HIV test and share the results with you?
1	1	1	1	Do you have HIV/AIDS
1	1	1	1	In which month and year, were you diagnosed with HIV/AIDS?
1	1	1	1	Yes or no? Were you on ARVs before this pregnancy?
1	1	1	1	In the last two months, did you ever miss taking your ARVs?
1	1	1	1	In the last two months, did you miss taking your ARVs, because the ARVs were out of stock at the health facility or pharmacy?
1	1	1	1	Did you miss taking your ARVs because you did not go to pick up the ARVs up at the health facility or pharmacy?
1	1	1	1	Did you miss taking your ARVs because you forgot to?
1	1	1	1	Did you skip taking your ARVs because the medication made you feel bad (side effects)?
0	0	1	1	Have you taken your baby for an HIV test?
0	0	1	1	Is your baby HIV positive or negative?

Section 6: Self Reporting Questionnaire (mental distress)				
**T1**	**T2**	**T3**	**T4**	**The following questions are related to certain pains and problems that may have bothered you in the last month (30 days). If you think the question applies to you and you had the described problem in the last 30 days, select Yes. On the other hand, if the question does not apply to you and you did not have the problem in the last 30 days, select No. If you are unsure about how to answer a question, please give the best answer you can.**
1	1	1	1	Do you often have headaches?
1	1	1	1	Is your appetite poor?
1	1	1	1	Do you sleep badly?
1	1	1	1	Are you easily frightened?
1	1	1	1	Do your hands shake?
1	1	1	1	Do you feel nervous, tense or worried?
1	1	1	1	Is your digestion poor?
1	1	1	1	Do you have trouble thinking clearly?
1	1	1	1	Do you feel unhappy?
1	1	1	1	Do you cry more than usual?
1	1	1	1	Do you find it difficult to enjoy your daily activities?
1	1	1	1	Do you find it difficult to make decisions?
1	1	1	1	Is your daily work suffering?
1	1	1	1	Are you unable to play a useful part in life?
1	1	1	1	Have you lost interest in things?
1	1	1	1	Do you feel that you are a worthless person?
1	1	1	1	Has the thought of ending your life been on your mind?
1	1	1	1	Do you feel tired all the time?
1	1	1	1	Do you have an uncomfortable feeling in your stomach?
1	1	1	1	Are you easily tired?

Section 7: ANC experience (positive pregnancy experience/satisfaction)				
**T1**	**T2**	**T3**	**T4**	**Now we will ask you about your experiences at antenatal clinic during this pregnancy. We ask you to use a scale from poor to excellent to rate some of your ANC experiences and the kind of care you received. Think about your entire ANC experience, then you will select a rating of poor, fair, good, very good or excellent.**
0	1	0	0	How do you rate how respectfully were you greeted at your ANC visits?
0	1	0	0	How do you rate how completely and carefully were you checked to make sure everything was okay at your ANC visits?
0	1	0	0	How do you rate the ANC midwives' knowledge and ability to take care of your health at your ANC visits?
0	1	0	0	How would you rate how well the exam procedures were explained to you at your ANC visits?
0	1	0	0	How would you rate the concern that midwives showed you when giving you care at ANC?
0	1	0	0	How would you rate how respectfully you were treated by midwives at ANC?
0	1	0	0	How would you rate the helpfulness of the advice that you got about keeping you and your baby healthy at ANC?
0	1	0	0	How would you rate the amount of personal attention you got from a midwife at ANC?
0	1	0	0	How do you rate ANC midwives' ability to listen to you at your ANC visits?
0	1	0	0	How would you rate the overall quality of your entire ANC experience?
0	1	0	0	Thinking about all your ANC visits, were you ever asked by any staff at the health facility for a bribe?
0	1	0	0	In general, did you spend more than an hour of your time waiting for services at ANC?
0	1	0	0	Which type of antenatal care did you attend? Were you in a group with other women throughout or pregnancy or were you in regular ANC?
0	1	0	0	If you got pregnant again, which type of ANC would you prefer?

				**Section 8: ANC Services and Content**
**T1**	**T2**	**T3**	**T4**	**Now you will hear questions about 10 specific services that you may have received at ANC during this pregnancy.**
0	1	0	0	Was your height measured at least once at ANC?
0	1	0	0	Was your weight measured at every ANC visit?

0	1	0	0	Was your blood pressure measured at every ANC visit?
0	1	0	0	Did the midwife listen for the baby's heart rate at every ANC visit?
0	1	0	0	Did the midwife check the position of the baby at every ANC visit?
0	1	0	0	Were you told how many weeks pregnant you were or **your** due date at every ANC visit?
0	1	0	0	At ANC, were you given iron tablets?
0	1	0	0	At ANC, were you give medications for intestinal worms?
0	1	0	0	At ANC, were given any medications to prevent malaria?
0	1	0	0	At ANC, did you get an injection to prevent the baby from getting tetanus, that is convulsions, after birth?
**Next you will be asked if you learned about various topics while at ANC.**				
0	1	0	0	Did you learn about how to eat healthier during pregnancy?
0	1	0	0	Did you learn about the danger signs of pregnancy?
0	1	0	0	Did you learn how to tell when you were in labor?
0	1	0	0	Did you learn about what to expect during childbirth?
0	1	0	0	Were you advised about making a plan to get to the health facility when you go into labor?
0	1	0	0	Did you learn about how to prevent HIV and other sexually transmitted diseases during sex?
0	1	0	0	Did you learn about ways to prevent malaria?
0	1	0	0	Did you learn about the importance of breastfeeding shortly after giving birth?
0	1	0	0	Did you learn about the importance of exclusive breastfeeding?
0	1	0	0	Did you learn that if your first HIV test is negative, you should get a second one in late pregnancy?
0	1	0	0	Did you learn about depression?
0	1	0	0	Did you learn that spacing births is important for your health and the health of your other children?
0	1	0	0	Did you learn about family planning methods?

**Section 9: ANC Community Building**				
**T1**	**T2**	**T3**	**T4**	**Question**
0	1	1	1	While at ANC, did you make new friends?
0	1	1	1	How many new friends?
0	1	1	1	Do you talk to any of these friends on the phone?
0	1	1	1	Do you visit with any of these friends?
**This section will focus on relationships you developed with other women while attending clinic. First, you will hear a list of words that are about the kinds of emotional support that women sometimes get from one another. As you hear each word, please indicate if you experienced this type of support from other pregnant women at ANC.**				
0	1	0	0	Love/Affection
0	1	0	0	Trust
0	1	0	0	Acceptance
0	1	0	0	Encouragement
0	1	0	0	Caring
0	1	0	0	Understanding
0	1	0	0	Respect
**Now you will hear different ways that women relate to one another. As you hear each word, please indicate if you experienced this with other women at ANC.**				
0	1	0	0	Companionship
0	1	0	0	Belonging
0	1	0	0	Shared activities
0	1	0	0	Shared experience
**The next set of words are about exchanging information. As I read each word, please tell me if you rechanged information in these ways**				
0	1	0	0	Exchanged advice
0	1	0	0	Exchanged guidance/suggestions
0	1	0	0	Exchange useful information

**Section 10: Pregnancy Related Empowerment Scale**

T1	T2	T3	T4	The following questions are in reference to your antenatal care and interactions with midwives at antenatal care. Please listen to each statement carefully and then choose the response that best describes how strongly your agree or disagree with the statement.
0	1	0	0	I can ask my midwife provider about my pregnancy.
0	1	0	0	I have enough time with my midwife to discuss my pregnancy.
0	1	0	0	My midwife listens to me.
0	1	0	0	My midwife respects me.
0	1	0	0	I expect my midwife to respect my decisions about my pregnancy.
0	1	0	0	My midwife respects my decision, even if it is different than her/his recommendation.
0	1	0	0	I take responsibility for the decisions I make about my pregnancy like eating healthy food.
0	1	0	0	I can tell when I have made a good health choice.
0	1	0	0	Since I began prenatal care, I have been making more decisions about my health.
0	1	0	0	Women need to share experiences with other women when they are pregnant.
0	1	0	0	I share my feelings and experiences with other women.
0	1	0	0	I know if I am gaining the right amount of weight during my pregnancy.
0	1	0	0	I have a right to ask questions when I don't understand something about my pregnancy.
0	1	0	0	I am able to change things in my life that are not healthy for me.
0	1	0	0	I am doing what I can to have a healthy baby.
0	1	0	0	If something is going wrong in my pregnancy, I know who to talk to.

**Section 11: Birth & Infant Information**				
**T1**	**T2**	**T3**	**T4**	**The next set of questions are about your most recent birth.**
0	0	1	0	On what date did you give birth?
0	0	1	0	Did you go into labor before reaching 38 weeks of pregnancy? In other words, was your baby born early?
0	0	1	0	Where did you deliver your baby?
0	0	1	0	Did you have a caesarean section?
0	0	1	0	Did you have excessive bleeding or a post-partum hemorrhage?

**Section 12: Infant(s)**				
**T1**	**T2**	**T3**	**T4**	
0	0	1	1	How many babies did you give birth to?
**The following questions are about your baby. If you had more than one baby, we will repeat the questions for each. But for now, think about the first baby born.**				
0	0	1	1	Is this baby living?
0	0	1	1	We are so sorry to hear that your baby died, on what date did your baby die?
0	0	1	1	Can you tell us what you think caused your baby's death?
0	0	1	1	Is this baby male or female?
0	0	1	0	How much did your baby weigh at birth?
0	0	1	0	When your baby was born, was he/she very large, larger than average, average, smaller than average, or very small?
0	0	1	0	Did you breastfeed this baby at all?
0	0	1	0	Did you experience any problems with breastfeeding your baby?
0	0	1	0	After you gave birth to your baby, how long was it before you put the baby to the breast for the first time?
0	0	1	0	Before breastfeeding your baby for the very first time, did you feed him/her anything else (water, tea, milk, etc.)?
0	0	1	0	In the first three days after giving birth, was your baby fed anything to drink other than breast milk?
0	0	1	0	Did you feed your baby colostrum?
0	1	1	0	How many months do you plan to exclusively breastfeed your baby?
0	0	0	1	How many months did you exclusively breastfeed your baby?
0	0	1	0	Has your baby had foods or drinks besides breast milk?
0	0	1	0	Does your baby eat or drink something other than breast milk **every day**?
**The next set of questions are about how you have fed your infant. Listen to this list of foods. Please mark all the foods that you baby has tried. Did you baby eat or drink the following?**				

0	0	1	1	Animal milk (e.g., cow or goat, etc.)
0	0	1	1	Plain water
0	0	1	1	Soft or fermented porridge
0	0	1	1	Mashed bananas
0	0	1	1	Tea
0	0	1	1	Fruit juice
0	0	1	1	Sugar or glucose water
0	0	1	1	Honey
0	0	1	1	Soda
0	0	1	1	Infant formula
0	0	1	1	Has your baby received any vaccinations?
0	0	1	1	Since your baby was born, how many times have you taken your baby to well-baby clinic?
0	0	1	1	What was the date of the baby's last checkup?
0	0	1	1	How much did the baby weigh at the last checkup?
**The next questions are about illnesses that this baby may have had that caused the baby to be hospitalized. Select Yes or No to tell us if this baby was hospitalized for.**				
0	0	1	1	Injury or accident
0	0	1	1	Cord infection
0	0	1	1	Diarrhea
0	0	1	1	Respiratory infection
0	0	1	1	Fever

**Section 13: PNC & Family Planning**				
**T1**	**T2**	**T3**	**T4**	**This is the last section of the survey. It is about family planning.**
1	0	0	0	Have you ever used any form of family planning before?
0	0	1	0	After the birth of your baby, did you get an operation to avoid having any more children?
0	0	1	0	Are you currently using any form of family planning to delay or prevent pregnancy?
0	0	1	1	Which method are you currently using to delay or prevent pregnancy?
0	0	1	1	Are you using condoms to delay or prevent pregnancy?
