# Supplementary material for: An effectiveness-implementation hybrid type 1 trial assessing the impact of group versus individual antenatal care on maternal and infant outcomes in Malawi
Source: BMC Public Health. 2020 Feb 10;20:205. doi: 10.1186/s12889-020-8276-x (PMC7008527; doi:10.1186/s12889-020-8276-x)
Supplement: Supplementary file 2 — Additional file 2: Fidelity and Self-Evaluation Forms [file 12889_2020_8276_MOESM2_ESM.pdf]

Fidelity Observation Form: Implementation Team

1. SESSION INFORMATION									
1A.	Clinic Name								
1.1	Date (dd/mm/yy)								
1.2	Observer								
1.3	What time was group scheduled to start?								
1.4	What time did the **first** woman arrive?								
1.5	What time did the **last** woman arrive?								
1.6a	What is this Group's number?								
1.6b	What is this Group's name?								
1.7	Meeting number	□ 2	□ 3	□ 4	□ 5	□ 6	□ 7	□ 8	□ PP
1.8	Name of midwife								
1.9	Name of volunteer								

2. PREPARATION			
2.1	Was the room set up before the women arrived?	□ **YES**	□ **NO**

3. GREETING AND SOCIALIZING			
3.1	Were women were greeted by the co-facilitators?	□ **YES**	□ **NO**
3.2	Are women socializing with one another?	□ **YES**	□ **NO**

4. HEALTH ASSESSMENTS			
4.1	What time did health assessments begin?		
4.2	Was self-assessment equipment available and functioning?	□ **YES**	□ **NO**
	**4.2a. If NO**, explain:		
4.3	Did <u>**all**</u> women participate in self-measures?	□ **YES**	□ **NO**
4.4	Did health assessments with the midwife take place within group space?	□ **YES**	□ **NO**
4.5	What time did health assessments end?		
4.5	Observations (note major challenges and/or successes of health assessments):		

5.0 GROUP HEALTH PROMOTION DISCUSSION			
5.1	What time did group discussion begin?		
5.2	Did discussion begin **after all** health assessments were completed?	□ **YES**	□ **NO**
	**5.2a. If NO**, explain:		

5.3	How many facilitators are present?		
5.4	How many women are present?		
5.5	How many partners are present?		
5.6	How many observers are present?		
5.7	Was discussion interrupted for any reason?	□ **YES**	□ **NO**
5.8	Did any of the participants arrive **after** group discussion already began?	□ **YES**	□ **NO**
5.9	Did anything challenging happen during this discussion?	□ **YES**	□ **NO**
	**5.9a.** If **YES**, please describe:		
5.13	What time did group discussion end?		

**6.0 ENVIRONMENT AND LOGISTICS**			
6.1	Is the room size adequate?	□ **YES**	□ **NO**
6.2	Were materials needed for activities present and in good repair?	□ **YES**	□ **NO**
6.3	Did both co-facilitators have a facilitator's guide available to them?	□ **YES**	□ **NO**
6.4	Was discussion conducted in a circle?	□ **YES**	□ **NO**
6.5	Did co-facilitators sit across from (not next to) one another in the circle?	□ **YES**	□ **NO**
6.6	Was there enough privacy?	□ **YES**	□ **NO**
6.7	Was everyone in the group able to hear?	□ **YES**	□ **NO**

**7. GROUP DYNAMICS**					
**About how many members engaged in the following?**		**None**	**One to a few**	**About half**	**Most to All**
7.1	Asked questions freely	□	□	□	□
7.2	Shared ideas, personal feelings, experiences	□	□	□	□
7.3	Were engaged, focused and participating	□	□	□	□
7.4	Avoided or resisted participating	□	□	□	□
7.5	Talked excessively and interrupted flow of discussion	□	□	□	□
7.6	Disrespectful or judgmental of other group members	□	□	□	□

**8. CO-FACILITATOR BEHAVIORS**					
		**Not at all**	**Partly**	**Mostly**	**N/A**
8.1	Facilitators were well-prepared	□	□	□	□

**8.2**	Facilitators used the guide well (did not just read)	☐	☐	☐	☐
**8.3**	Facilitators worked collaboratively	☐	☐	☐	☐
**8.4**	Facilitators actively used acknowledge, refer and return (ARR)	☐	☐	☐	☐
**8.5**	Anything important to note about facilitator interactions with one another?				

**9. FACILITATION**		
**9.1**	Overall, to what extent was this group meeting more like a class or more like a discussion?	☐ Mostly like a class<br>☐ More a class than a discussion<br>☐ Half class, half discussion<br>☐ More a discussion than a class<br>☐ Mostly like a discussion
**9.2**	Overall, what was the level of engagement and connectedness among group members throughout this session?	☐ Low<br>☐ Low to medium<br>☐ Medium<br>☐ Medium to high<br>☐ High
**9.3**	Is there anything else important to document about this meeting?	

**10. SELF-EVALUATION REVIEW**	
**10.1**	How did you feel about today?
**10.2**	What went well?
**10.3**	What could be improved?

11. CONSTRUCTIVE FEEDBACK

- Before you share your feedback, ask if they would like to hear your comments from your observations.
- Tactfully describe strengths and areas for improvements that you noted. ALWAYS begin with at least one strength.
- End with a positive suggestion that would help them to improve their facilitation skills.
- Make note of the feedback you offered to the facilitators, noting if responses to the feedback were positive (accepting), neutral (no response) or negative (defensive).

11.1 Feedback Notes:

Care Circles: Co-facilitators Self Evaluation Fidelity Form								
**Date**								
**Facilitators**								
**Clinic name**								
**Group number**								
**Group name**								
**Meeting**	**2**	**3**	**4**	**5**	**6**	**7**	**8**	**PP**

Part A

**1.** Did Health assessments take place in group space?	☐ **YES**	☐ **NO**
**2.** Did **all** women participate in self-measures?	☐ **YES**	☐ **NO**
**3.** Did discussion take place in a circle?	☐ **YES**	☐ **NO**
**4.** Was the group size 8-12 women only?	☐ **YES**	☐ **NO**
**5.** Was at least one **assigned** co-facilitator present?	☐ **YES**	☐ **NO**
**6.** Were women were socializing with one another?	☐ **YES**	☐ **NO**
**7.** Did women talk freely and share experiences?	☐ **YES**	☐ **NO**

Part B

**8.** To what extent was the session like a class or like a discussion?	☐ **Mostly like a class**<br>☐ **Somewhat like a class**<br>☐ **Half class, half discussion**<br>☐ **Somewhat a discussion**<br>☐ **Mostly a discussion**

Part C

**9.** Overall, how engaged and connected were group members at this session?	☐ **Very Low**<br>☐ **Low to medium**<br>☐ **Medium**<br>☐ **Medium to high**<br>☐ **Very High**

12. What can be done to improve your facilitation skills?

13. What can be done to improve this session's activities?

14. Describe ideas for new activities that might work for this or other sessions
