# Supplementary material for: An effectiveness-implementation hybrid type 1 trial assessing the impact of group versus individual antenatal care on maternal and infant outcomes in Malawi
Source: BMC Public Health. 2020 Feb 10;20:205. doi: 10.1186/s12889-020-8276-x (PMC7008527; doi:10.1186/s12889-020-8276-x)
Supplement: Supplementary file 3 — Additional file 3: Benchmark Scoring Sheet [file 12889_2020_8276_MOESM3_ESM.pdf]

The 3-Step Implementation Model Benchmarks
(Prepare, Rollout, and Sustain)

Group Care Circles Quarterly Benchmarks: PREPARE			
**Health Facility:**	**Today's date:**		
**Reporting Quarter (3 months):**	**Completed by:**		
**1.** Was the health facility sensitized to Group Care Circles?		☐ YES	☐ NO
**2.** Did co-facilitators (midwives/nurses and community volunteers, able to read Chichewa), attend a Care Circles training workshop?		☐ YES	☐ NO
**2a.** In total, how many midwives/nurses are available for Care Circles?			
**2b.** In total, how many community volunteers are available for Care Circles?			
**3.** Are administrators supportive of Group Care Circles?		☐ YES	☐ NO
**3a.** Describe the ways that administrators are and are not supportive:			
**4.** Did you meet with a KCN consultant to initiate implementation planning?		☐ YES	☐ NO
**5.** Was a Care Circles room identified and reserved?		☐ YES	☐ NO
**6.** Was a Care Circles coordinating team was formed?		☐ YES	☐ NO
**6a. In total**, how many people are on the team?			
**7.** Did the coordinating team met at least one time to create the rollout plan?		☐ YES	☐ NO
**7a. In total**, how many did the team meet?			
**8.** Was the rollout plan shared and approved by the health facility?		☐ YES	☐ NO
**9.** Did the coordinating team setup a Care Circles schedule that ensures continuity of care (the same co-facilitators at each group)?		☐ YES	☐ NO
**10.** Was the first Care Circles group established? In other words, were 8-12 women of similar gestational ages recruited and given the dates for all group meetings?		☐ YES	☐ NO
**10a.** What is the date of the first Care Circles meeting?			

Group Care Circles Quarterly Benchmarks: ROLLOUT		
**Health Facility:**	**Today's date:**	
**Reporting Quarter (3 months):**	**Completed by:**	
**A.** How many groups fully completed care (all meetings) **<u>this quarter</u>**?		
**B.** Did new clinic leaders & staff join the health facility? ☐ Yes ☐ No		
**1.** Are administrators supporting Group Care Circles?	☐ YES	☐ NO
**1a.** Describe the ways that administrators are or are not supportive:		
**2.** Is everyone, including the new leaders and staff, sensitized to Care Circles?	☐ YES	☐ NO
**3.** Does your health facility have adequate numbers of trained co-facilitators?	☐ YES	☐ NO
**5a. In total**, how many midwives/nurses are available for Care Circles?		
**5b. In total**, how many community volunteers are available for Care Circles?		
**5c. In total**, how many **new** facilitators were trained **this quarter**?		
**4.** Did the coordinating team meet at least once **this quarter**?	☐ YES	☐ NO
**6a. In total**, how many times did the team meet **this quarter**?		
**5.** Did you make plans to rollout new groups **this quarter**?	☐ YES	☐ NO
**7a.** How many new groups did you **plan** to offer **this quarter**?		
**7b.** Is this number more, the same, or less than last quarter?		
**6.** Did you form new ANC Care Circles **this quarter**?	☐ YES	☐ NO
**8a. In total**, how many **new** ANC Care Circles were formed?		
**8b.** If no new groups were formed, **please explain**:		
**7.** Did any Care Circles end prematurely **this quarter**?	☐ YES	☐ NO
**9a.** How many Care Circles ended prematurely?		
**9b.** If Care Circles ended prematurely, **please explain:**		
**8.** Did coordinators collect and review self-assessment forms?	☐ YES	☐ NO
**8a.** How many self-evaluation forms were collected?		
**8b.** How many forms have a score above **13**?		
**8c. Calculate the percentage of forms with a score above 13 (8b ÷ 8a)**		
**8d.** How many self-evaluation forms are missing?		
**9.** Were Care Circles updates presented regularly at health facility meetings?	☐ YES	☐ NO
**10.** Does your health facility plan to continue offering Care Circles?	☐ YES	☐ NO

Group Care Circles Quarterly Benchmarks: SUSTAINING		
**Health Facility:**	**Today's date:**	
**Reporting Quarter (3 months):**	**Completed by:**	
**A.** How many groups fully completed care (all meetings) **this quarter**?		
**1.** Is everyone, including the new leaders and staff, sensitized to Care Circles?	☐ YES	☐ NO
**2.** Does your health facility have adequate numbers of trained co-facilitators?	☐ YES	☐ NO
**2a. In total**, how many midwives/nurses are available for Care Circles?		
**2b. In total**, how many community volunteers are available?		
**2c. In total**, how many **new** facilitators were trained **this quarter**?		
**3.** Did you roll out new groups **this quarter**?	☐ YES	☐ NO
**3a. If NO**, please explain:		
**4.** Did coordinators collect and review self-assessment forms?	☐ YES	☐ NO
**4a.** How many self-evaluation forms were collected?		
**4b.** How many forms had a score above 13.0?		
**4c.** How many self-evaluation forms are missing?		
**4d. Calculate** the percentage of forms with a score above 13.0 **(8b ÷ 8a)**		
**4e. See 4d,** were 90% (or more) of groups offered with high fidelity?		
**5.** Will your health facility continue to offer Group ANC Care Circles and enroll women in new groups?	☐ YES	☐ NO
